# Investigating Conceptual Models for the Relationship Between Depression and Condomless Sex Among Gay, Bisexual, and Other Men Who have Sex with Men: Using Structural Equation Modelling to Assess Mediation

**DOI:** 10.1007/s10461-019-02724-0

**Published:** 2019-11-28

**Authors:** A. R. Miltz, A. J. Rodger, A. Cozzi Lepri, J. Sewell, N. C. Nwokolo, S. Allan, C. Scott, D. Ivens, M. Lascar, A. Speakman, A. N. Phillips, L. Sherr, S. Collins, J. Elford, F. C. Lampe, for the AURAH Study Group

**Affiliations:** 1 Institute for Global Health, University College London, London, UK; 2 56 Dean Street, London, UK; 3 City of Coventry Healthcare Centre, Coventry, UK; 4 West London Centre for Sexual Health, London, UK; 5 Royal Free Hospital, London, UK; 6 Whipps Cross Hospital, London, UK; 7 HIV i-Base, London, UK; 8 City, University of London, London, UK

**Keywords:** Gay, bisexual and other men who have sex with men (MSM), Sexual behaviour, Depression, Structural equation modelling

## Abstract

The aim of this study is to investigate five hypothesized mechanisms of causation between depression and condomless sex with ≥ 2 partners (CLS2+) among gay, bisexual, and other men who have sex with men (GBMSM), involving alternative roles of self-efficacy for sexual safety and recreational drug use. Data were from the AURAH cross-sectional study of 1340 GBMSM attending genitourinary medicine clinics in England (2013–2014). Structural equation modelling (SEM) was used to investigate which conceptual model was more consistent with the data. Twelve percent of men reported depression (PHQ-9 ≥ 10) and 32% reported CLS2+ in the past 3 months. AURAH data were more consistent with the model in which depression was considered to lead to CLS2+ indirectly via low self-efficacy for sexual safety (indirect Beta = 0.158; p < 0.001) as well as indirectly via higher levels of recreational drug use (indirect Beta = 0.158; p < 0.001). SEM assists in understanding the relationship between depression and CLS among GBMSM.

## Introduction

In 2017, there were 4363 new HIV diagnoses in the UK of which 53% were reported among gay, bisexual, and other men who have sex with men (GBMSM) [1]. Although there is evidence that new HIV diagnoses are declining in the UK, especially in large clinics in London [2], GBMSM remain the group most at risk of HIV infection. From 2014 to 2017 there has been a 61% increase in diagnoses of chlamydia and syphilis and 43% increase in diagnoses of gonorrhoea among GBMSM. While this may be due, in part, to better detection of these STIs and/or behavioural changes possibly as a result of dating apps and pre-exposure prophylaxis (PrEP) use [3], the increased gonorrhoea incidence is of particular concern given that a number of antimicrobial resistant strains have recently been identified in the UK [3]. Accordingly, efforts to better understand and address condom use among GBMSM remain vital [4].

There is substantial evidence for an association between symptoms of depression and increased engagement in condomless sex (CLS), including CLS with multiple partners and partners of an unknown/sero-different HIV status [5–21], and HIV acquisition [22] in studies of GBMSM. Most of these studies have been conducted in the U.S. To date, two UK cross-sectional studies have investigated the relationship between symptoms of depression and CLS. In a 1999 genitourinary medicine (GUM) clinic sample of 122 GBMSM, a higher prevalence of depressive symptoms (HADS score ≥ 11) was observed among men who reported CLS with an HIV-positive/unknown status partner [7]. The AURAH (*Attitudes to and Understanding of Risk of Acquisition of HIV*) study collected data from individuals attending 20 GUM clinics in England from 2013 to 2014. Among a sub-sample of 1340 GBMSM, depressive symptoms were found to have moderate to strong associations with measures of CLS in the past 3 months, and with bacterial STI diagnosis and post-exposure prophylaxis (PEP) use in the past year [21]. Associations remained after adjusting for socio-demographic factors and additionally adjusting for smoking, higher-risk drinking, and recreational drug use [21]. Although cross-sectional studies have provided strong evidence for a link between depression and CLS, they cannot provide insight into the direction of association.

There are two established theories of the mechanisms by which depression may lead to sexual risk taking: social cognitive theory and cognitive escape theory. Social cognitive theory postulates that depression negatively impacts on one’s self-efficacy i.e. perceived capacity to influence life events and social conditions. Following a social cognitive perspective on behavioural management, the main requirement for effective sexual risk reduction is perceived self-efficacy to achieve sexual safety [23]. There are a number of psychosocial factors that have been hypothesized to lower one’s self-efficacy for sexual safety; drug and alcohol use, experiences of sexual coercion/abuse, and most commonly, depression [24, 25]. Cognitive escape theory suggests that some individuals may respond to threatening cues, such as risk of STI/HIV transmission, by escaping rational self-awareness [26]. Those who experience depressive symptoms may be more likely to engage in escapism as a coping strategy, characterised by fatalistic beliefs such as helplessness or powerlessness in the face of the HIV threat [27]. Such escapism may be as a result of feeling ill-equipped to mitigate the potential threat and may lead to risk taking [28, 29]. Substance use has consistently been linked to CLS [30, 31], and may be one of the key strategies used to facilitate a state of cognitive escape [32, 33].

Therefore, the mechanism of causation for the often-found link between depression and CLS may involve causal pathways via self-efficacy for sexual safety and/or recreational drug use, or via both of these factors on the same causal chain. These relationships are complicated further by the bidirectional nature of the association between depression and recreational drug use, and between self-efficacy for sexual safety and recreational drug use. Drug use may be a confounder or mediator of the association of depression with CLS, and of self-efficacy with CLS. Self-efficacy may be a confounder or mediator of the association of drug use with CLS.

Mediation analysis investigates whether the association between an exposure and outcome operates fully or partially via an intermediate factor(s) within a hypothesized causal chain [34]. Very few studies have tested mechanisms explaining the link between depressive symptoms and CLS using mediation analysis. One such study has been conducted among U.S. GBMSM (using baseline data from Project MIX, N = 1540, 2004–2006) [14]. Measures of both low self-efficacy for sexual safety and cognitive escape tendencies were found to mediate the cross-sectional association between depression and CLS with partners of unknown/HIV sero-different status in the past 3 months. In all analyses, age, income, education level, ethnicity, HIV status, and research site were adjusted for. A recent U.S. longitudinal study of GBMSM has also examined self-efficacy for sexual safety as a mediator of the relationship between number of ‘syndemic’ factors (occurrence of depression, childhood sexual abuse, intimate partner violence, substance use, and/or sexual compulsivity) and CLS with casual partners in the past 3 months [35]. At baseline (N = 197), a greater number of ‘syndemics’ was associated with CLS indirectly via low self-efficacy, but this finding was not entirely supported when investigating a longitudinal model, as changes in self-efficacy were not associated with changes in CLS over time. Finally, in a recent study of Canadian GBMSM (N = 703), poly drug use (use of three or more recreational drugs in the past 6 months) partially mediated the relationship between depression and CLS with partners of unknown/HIV sero-different status in the past 6 months [36].

There is a lack of research exploring the role of self-efficacy for sexual safety and recreational drug use on the causal pathway leading from depression to sexual risk behaviour. The aim of this study is to use data from the aforementioned AURAH study to examine and compare five conceptual models by which depression may lead to sexual risk taking. Each model has a different hypothesized mechanism of causation involving either self-efficacy or drug use, or both factors, on separate or combined causal pathways. These causal mechanisms are investigated within a conceptual model of causal connections between demographic factors, socio-economic factors, psychosocial measures, and sexual risk behaviour among GBMSM. This will be achieved using mediation analysis within the framework of structural equation modelling (SEM). SEM does not circumvent the issue that causal effects cannot be established in observational cross-sectional studies. However, it does allow for specification of relationships between variables, and of more complex relationships that distinguish between direct and indirect effects [37]. The advantage of SEM is the ability to understand the relationship between depression and CLS within the context of the hypothesized precursors of depression. This has implications for informing complex interventions that encompass targeting of multiple behaviours/social conditions along causal chains, in order to more effectively reduce/prevent sexual risk taking.

## Methods

The AURAH study recruited individuals aged 18 years or over without diagnosed HIV who were attending one of 20 GUM clinics in England from June 2013 to November 2014 [38]. A total of 4380 eligible patients were approached over the study period of whom 2630 self-completed a confidential questionnaire (response rate was 60%), which collected information on demographic, socio-economic, psychosocial, health, and lifestyle factors.

In total, 1484 men were classified as GBMSM as they met at least one of the following criteria: (i) reported being gay or bisexual (including other plurisexual identity labels i.e. identities that are not explicitly based on attractions to one sex/gender [39]), (ii) reported anal sex with a man in the past 3 months, or (iii) reported having disclosed to their family, friends or workmates as being gay, bisexual and/or attracted to men. Men who did not report any sex (anal or vaginal) in the past 3 months were excluded from this analysis since this is a study of sexually active men.

### Devising a Conceptual Model of Causal Connections Between Socio-economic Factors, Psychosocial Measures, and Sexual Risk Behaviour Collected in the AURAH Study; with Various Mechanisms of Causation Between Depression and Sexual Risk Behaviour

The following demographic/socio-economic and psychosocial factors collected in AURAH, which were identified as being relevant to depression and/or CLS among GBMSM, were investigated in this study: age (as a continuous variable), born in the UK (yes or no/missing), financial security i.e. enough money to cover basic needs (all of the time, most of the time, some of the time, or never), level of educational attainment (no qualifications, O levels/GCSEs, A levels, vocational qualifications, or university degree or higher), ongoing relationship with a partner (yes or no/missing), frequency of attending gay cafes, pubs, bars, nightclubs/discos or saunas (two or more times a month or less than twice a month), and concealment of sexual identity i.e. proportion of close family, friends and workmates who know that you are gay, bisexual and/or attracted to men (more than a few/missing or few/none). AURAH participants were also asked to report whether they had used recreational drugs in the past 3 months and, if so, to select which drug or drugs from the following list of 19 options; acid, anabolic steroids, cannabis, cocaine, crack, codeine, methamphetamine, MDMA, GHB/GBL, heroin, ketamine, khat, mephedrone, morphine, opium, poppers, speed, erectile dysfunction drugs, or other drug. The number of individual drugs used in the past 3 months (continuous variable) was the measure investigated in this study, given the hypothesis that vulnerability to depression and the likelihood of engaging in CLS [40] would increase with increasing numbers of drugs used.

A number of survey items were used to investigate depressive symptoms, self-efficacy for sexual safety, and levels of a supportive network. The statistical handling of these variables in SEM is described below. Depressive symptoms were measured using the Patient Health Questionnaire (PHQ-9) [41], a standardised inventory that inquires about frequency of occurrence of nine symptoms during the previous 2 weeks. Response options include: not at all (coded as 0), several days (coded as 1), more than half the days (coded as 2), and nearly every day (coded as 3). The PHQ-9 aims to provide a diagnostic measure of a chronic mental health condition, therefore, it is not unreasonable to assume that for many participants, symptoms may have preceded (sexual) behaviours in the past 3 months. The following statements were used to measure self-efficacy for sexual safety: (i) ‘I feel confident that, if I want to, I can make sure a condom is used during sex with any partner, in any situation’, and (ii) ‘I find it difficult to discuss condom use with any new sexual partner’. Response options included: strongly agree, tend to agree, undecided/no opinion/not relevant to me, tend to disagree, and strongly disagree. Responses were coded as 1 to 5, starting with ‘strongly agree’ (coded as 1) for the first statement and ‘strongly disagree’ (coded as 1) for the second statement. A high score amounts to low self-efficacy for both items. Levels of a supportive network were measured using a modified version of the Duke-UNC Functional Social Support Questionnaire [42]. This scale enquired about the level (as much as I would like, almost as much as I would like, some, but would like more, less than I would like, or much less than I would like) at which participants received the following support from other people: ‘I have people who care what happens to me’, ‘I get love and affection’, ‘I get chances to talk to someone I trust about my personal problems’, ‘I get invitations to go out and do things with other people’, and ‘I get help when I am sick in bed’. The response options were coded as 1 to 5, starting with ‘much less than I would like’ (coded as 1).

In terms of sexual behaviour, men were asked about anal sex with men, and anal or vaginal sex with women, in the past 3 months. Subsequent questions asked about CLS, including the number of partners with whom they had CLS (with response options; 1, 2–4, 5–10, or > 10). In this study, CLS with ≥ 2 partners (yes or no/missing) in the past 3 months, regardless of the gender of the partner, was investigated. This measure may confer more significant risk of STI/HIV infection than CLS with one partner, and would exclude men having CLS only with a stable partner.

A conceptual model of hypothesized causal relationships between all factors described above was devised. Of note, implicit within the conceptual model is that CLS is often a desirable behaviour for reasons of intimacy and pleasure. Sex with a new or non-regular partner may involve a perceived risk of STIs/HIV that, for some individuals, results in behavioural adaptation via condom use. Hypothesized causal relationships were derived from previous findings of other research studies (described in the discussion section), although such relationships have not been proven to be causal. Findings from the AURAH study were not used to inform hypothesized relationships. This includes previously published data on correlates of depression [21]. The postulated relationships are discussed in the context of the results of the model.

The conceptual model includes a number of hypothesized mechanisms of causation between depression and CLS with ≥ 2 partners: (i) low self-efficacy for sexual safety as the mediator (and recreational drug use as a confounder), (ii) recreational drug use as the mediator, (iii) both low self-efficacy and recreational drug use as mediators on two separate pathways, (iv) low self-efficacy as a mediator leading to recreational drug use as a mediator on the same pathway, and (v) recreational drug use as a mediator leading to low self-efficacy as a mediator on the same pathway.

### Statistical Analysis

This analysis used SEM to explore the consistency of the AURAH data with five conceptual models incorporating different hypothesized mechanisms of causation between depression and CLS with ≥ 2 partners.

Depressive symptoms, self-efficacy for sexual safety, and a supportive network were considered to be unobserved (latent) variables in SEM. Latent variables are hypothetical constructs that attempt to measure real phenomena but cannot be directly measured, as they are created in order to explain observed variation in behaviours, attitudes, and feelings, which researchers can measure (observable items) [43]. For depressive symptoms, the nine observable items on the PHQ-9 [41] were used to define the construct of depression. For self-efficacy for sexual safety, two observable items (described above) were used. The five observable items on the Duke-UNC Functional Social Support Questionnaire (described above) were used to define the construct of a supportive network [42].

Three confirmatory factor analyses (CFAs) were incorporated into each SEM model. In CFA, for each individual, a predefined latent variable is constructed from the average response to the observed survey items in the scale, which are weighted by the regression coefficients (factor loadings) for the relationship between the common variance among observed items in the scale and each of the observed items. If the common variance is strongly associated with the variance of an observed item, the factor loading will be larger and this observed item would be given greater weight in the derivation of the latent variable. Conversely, the more ‘measurement error’ in the observed item (variability that is not shared among observed items in the scale) the less weight this item will be given when constructing the latent variable. As a result, measurement error in the observed items is taken into account in CFA and the latent variable can be treated as if it was measured without error. In this study, treating depression as a latent variable instead of using the existing PHQ-9 scoring system was considered appropriate in the context of SEM given that latent variables account for unreliability of measurement.

Mediator pathways were incorporated and assessed by specifying indirect pathways in SEM. It was investigated whether depression was associated with CLS with ≥ 2 partners indirectly through: model (i) low self-efficacy for sexual safety, model (ii) higher levels of recreational drug use, model (iii) low self-efficacy for sexual safety and separately via higher levels of recreational drug use, model (iv) low self-efficacy for sexual safety and then recreational drug use, and model (v) recreational drug use and then low self-efficacy for sexual safety. A significant indirect effect indicates that a hypothesized intermediate factor may be on the causal pathway [44].

In all five conceptual models, mediator pathways were also incorporated to investigate the following hypotheses: (i) concealment of sexual identity causes depression indirectly via less frequent gay venue attendance and lower levels of a supportive network, (ii) concealment of sexual identity leads to lower levels of recreational drug use via less frequent gay venue attendance, (iii) higher levels of educational attainment reduces risk of depression via financial security, and (iv) older age (and therefore greater exposure to harmful anti-gay norms and discriminatory laws) lowers levels of recreational drug use via concealment of sexual identity and less frequent visits to gay venues.

SEM is an extension of multiple linear regression modelling that provides estimates of the magnitude and significance of hypothesized causal connections between sets of variables. It is best depicted via use of a causal path diagram. A path coefficient is a standardized regression coefficient (Beta weight). The interpretive value extends only to that of comparing effect sizes in order to determine which factor is of ‘greater importance’ to the model; the greater the coefficient, the greater the importance. A positive coefficient indicates a positive relationship with the outcome variable and a negative coefficient indicates the opposite, an inverse relationship with the outcome variable. The p-values correspond to the estimates adjusted for their standard errors (equivalent to the z score). p-values < 0.05 are considered to indicate significant associations.

The comparative fit index (CFI), the tucker-lewis index (TLI), and the root mean square error of approximation (RMSEA) test were used to guide the conclusion as to the model fit. The model is considered to have a satisfactory fit if: CFI and TLI are ≥ 0.90 and RMSEA is ≤ 0.08. The model is considered to have a good fit if: CFI and TLI are ≥ 0.95 and RMSEA is ≤ 0.06 (higher 90% CI ≤ 0.08; p > 0.05) [37, 45]. Given the limitations of the χ^2^ test, this model fit index was not used [37]. Path coefficients presented in this paper are from the conceptual model with the best fit.

All analyses were performed using Mplus statistical software [46]. A generalized weighted least square based robust estimator (the mean and variance-adjusted WLS, WLSMV) was incorporated due to the inclusion of binary/categorical outcome variables.

## Results

### Descriptive Statistics

Of the 2630 individuals who participated in the AURAH Study, 1484 were classified as GBMSM. The current analysis is based on 1340 GBMSM who reported sex in the past 3 months. Of these, 1238 (92.4%) reported anal sex with men only, 66 (4.9%) reported sex with both men and women, and 36 (2.7%) reported sex with women only in the past 3 months [21]. Eighty-nine percent of men identified as gay (9.5% as bisexual/other plurisexual identity and 1.4% as straight) and 82.1% were of white ethnicity. Other socio-economic and psychosocial factors used in the analysis are shown in Table 1.

**Table 1:** Socio-economic and psychosocial characteristics of the sample

N = 1340 GBMSM who reported anal/vaginal sex (past 3 months)	n (%)
Age (continuous variable)	
Median (IQR, 25–75%)	31 (26–39)
Age groups (years)	
18–24	235 (17.8%)
25–29	344 (26.0%)
30–34	255 (19.3%)
35–39	175 (13.2%)
40–44	125 (9.5%)
45–49	93 (6.9%)
50 +	95 (7.1%)
Born in the UK	
Yes	762 (56.9%)
No/missing^a^	578 (43.1%)
Money to cover basic needs	
Always	958 (71.7%)
Mostly	281 (21.0%)
At times	70 (5.2%)
Never	27 (2.0%)
Level of educational attainment	
No qualifications	34 (2.6%)
O levels/GCSEs	120 (9.1%)
A levels	240 (18.1%)
Vocational qualifications	41 (3.1%)
University degree or higher	891 (67.2%)
Ongoing relationship with a partner	
Yes	579 (43.2%)
No/missing^a^	761 (56.8%)
Number of recreational drugs used (past 3 months) (continuous variable)	
Median (IQR, 25–75%)	2 (1–4)
Number of recreational drugs used (past 3 months)	
0	568 (42.7%)
1	269 (20.2%)
2	151 (11.4%)
3	88 (6.6%)
4	63 (4.7%)
5 +	190 (14.3%)
Frequency of gay venue (gay cafes, pubs, bars, nightclubs/discos or saunas) visits	
Two or more times a month	684 (51.6%)
Less than twice a month	642 (48.4%)
Concealment of sexual identity: proportion of close family/friends/workmates who know you are gay/bisexual/attracted to men	
More than a few/missing^a^	1284 (95.8%)
Few/none	56 (4.2%)
Measure of supportive network: *I have people who care what happens to me*	
Less or much less than I would like	73 (5.5%)
Measure of supportive network: *I get love and affection*	
Less or much less than I would like	148 (11.1%)
Measure of supportive network: *I get chances to talk to someone I trust about my personal problems*	
Less or much less than I would like	154 (11.6%)
Measure of supportive network: *I get invitations to go out and do things with other people*	
Less or much less than I would like	125 (9.4%)
Measure of supportive network: *I get help when I am sick in bed*	
Less or much less than I would like	205 (15.4%)
PHQ-9 (1) Little interest or pleasure in doing things	
More than half or nearly every day	84 (6.4%)
PHQ-9 (2) Feeling down, depressed, or hopeless	
More than half or nearly every day	121 (9.2%)
PHQ-9 (3) Trouble falling or staying asleep, or sleeping too much	
More than half or nearly every day	224 (16.9%)
PHQ-9 (4) Feeling tired or having little energy	
More than half or nearly every day	207 (15.7%)
PHQ-9 (5) Poor appetite or overeating	
More than half or nearly every day	118 (8.9%)
PHQ-9 (6) Feeling bad about yourself-or that you are a failure or have let yourself or your family down	
More than half or nearly every day	169 (12.8%)
PHQ-9 (7) Trouble concentrating on things, such as reading the newspaper or watching television	
More than half or nearly every day	114 (8.6%)
PHQ-9 (7) Trouble concentrating on things, such as reading the newspaper or watching television	
More than half or nearly every day	114 (8.5%)
PHQ-9 (8) Moving or speaking so slowly that other people could have noticed/being so restless that it is hard to sit still	
More than half or nearly every day	21 (1.6%)
PHQ-9 (9) Thoughts that you would be better off dead, or of hurting yourself in some way	
Several, more than half or nearly every day	157 (11.9%)
Measure of self-efficacy for sexual safety: *I feel confident that, if I want to, I can make sure a condom is used during sex with any partner, in any situation*	
Strongly agree	900 (67.9%)
Tend to agree	342 (25.8%)
Undecided/no opinion/NA	36 (2.7%)
Tend to disagree	38 (2.9%)
Strongly disagree	9 (0.7%)
Measure of self-efficacy for sexual safety: *I find it difficult to discuss condom use with any new sexual partner*	
Strongly disagree	754 (57.0%)
Tend to disagree	346 (26.2%)
Undecided/no opinion/NA	81 (6.1%)
Tend to agree	90 (6.8%)
Strongly agree	52 (3.9%)

^a^Born in the UK: 2.1% (n = 28) missing. Ongoing relationship: 0.2% (n = 3) missing. Concealment of sexual identity: 3.0% (n = 40) missing

The prevalence of clinically significant depressive symptoms based on a common PHQ-9 cut-off score across the nine questions of ≥ 10, was 12.4% (n = 166/1340) [21], see Table 1 for individual PHQ-9 symptoms. Thirty-two percent of men did not strongly agree that they felt confident to ensure condom use (statement 1) and 10.7% tended to or strongly agreed that they had difficulty discussing condom use (statement 2), see Table 1. In total, 56.8% (n = 761) of men reported using one or more recreational drugs in the past 3 months (see Table 1 for number of drugs used). Further details of the use of specific drugs among GBMSM in the AURAH study have been published elsewhere [47]. Overall, 64% of men reported CLS with one or more partners and 32% (n = 430) reported CLS with ≥ 2 partners in the past 3 months. Of the 430 men who reported CLS with ≥ 2 partners, 90.2% (n = 388) had male CLS partners only, 6.0% (n = 26) had at least one male and at least one female CLS partner, and 3.7% (n = 16) had female CLS partners only.

### Conceptual Model of Causal Connections Between Socio-economic Factors, Psychosocial Measures, and Sexual Risk Behaviour Collected in the AURAH Study; with Various Mechanisms of Causation Between Depression and Sexual Risk Behaviour

**Fig. 1:**
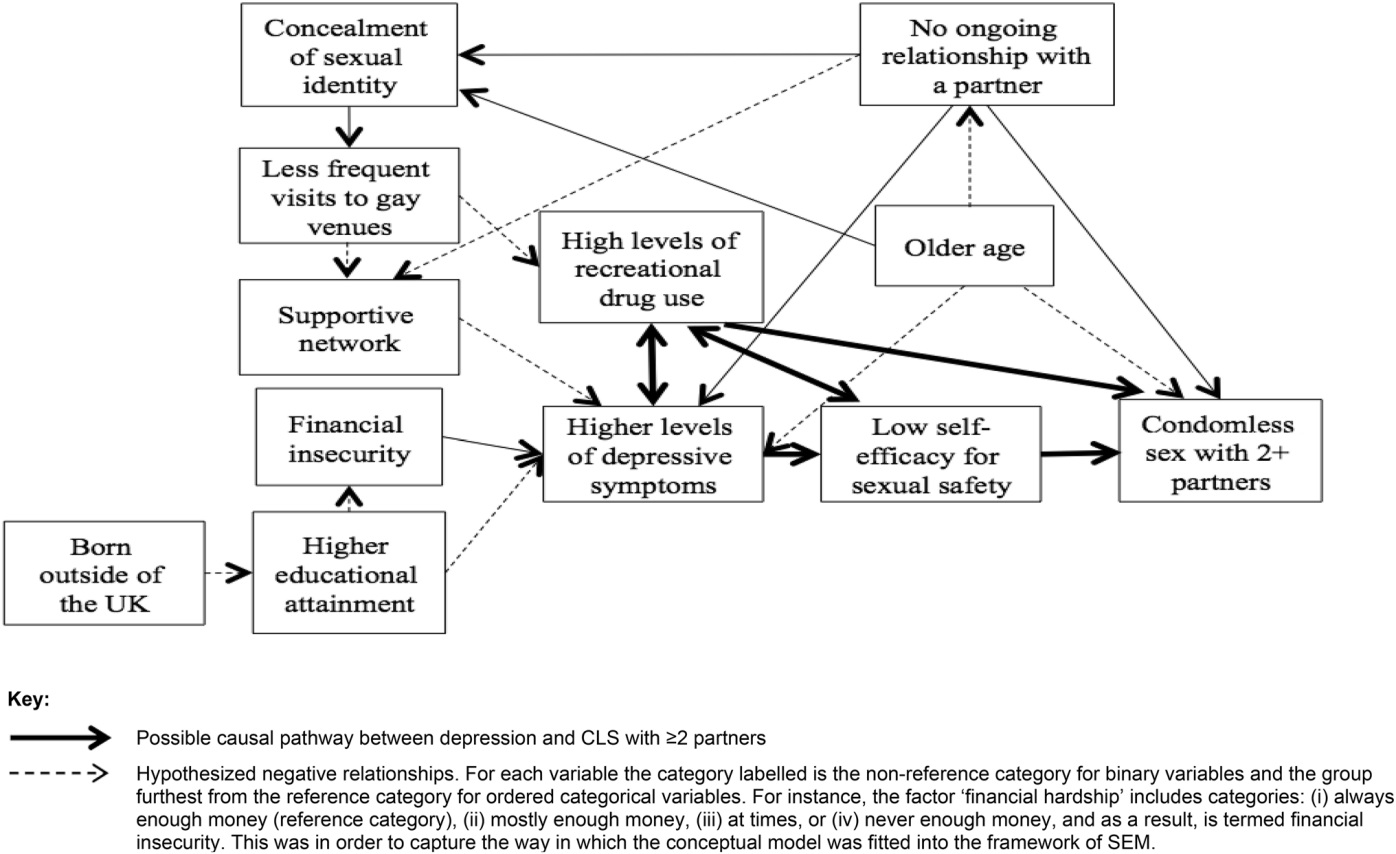
Overall conceptual model of hypothesized causal connections between socio-economic factors, psychosocial measures, and sexual risk behaviour collected in the AURAH study

Figure 1 presents the hypotheses made about causal connections between socio-economic factors, psychosocial measures, and sexual risk behaviour collected in the AURAH study. Factors are labelled according to how they were modelled in the SEM i.e. the non-reference category for binary variables and the group furthest from the reference category for ordered categorical variables is the category that is labelled. Inverse (negative) hypothesized relationships are indicated.

### Exploring Various Mechanisms of Causation Between Depression and CLS with ≥ 2 Partners

**Fig. 2:**
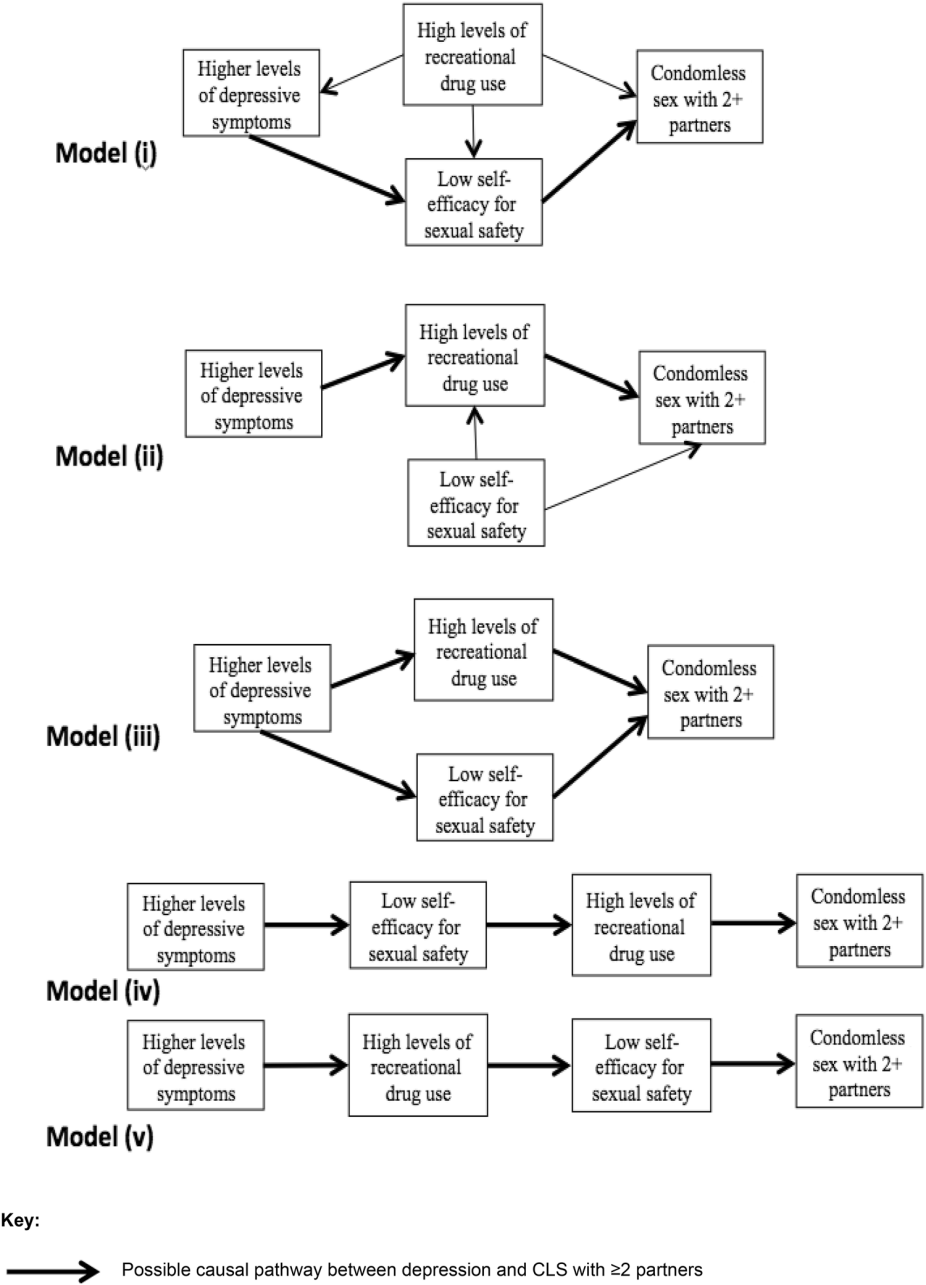
Specific causal mechanisms investigated in five separate conceptual models (arrows between other factors remain the same as in Fig. 1)

Figure 2 presents five mechanisms of causation between depressive symptoms and CLS with ≥ 2 partners, whereby the five alternative roles of self-efficacy for sexual safety and recreational drug use are considered. The indirect pathway(s) specified (arrows in bold in Fig. 2) was significant in all five models (p < 0.001 for all indirect effects examined). Table 2 presents the model fit indices. Conceptual models (i), (iii), and (iv) appeared more consistent with the data as the *p* value for the RMSEA close-fit test was > 0.05. In model (i) depression was associated with CLS with ≥ 2 partners indirectly through low self-efficacy for sexual safety (indirect Beta = 0.158 [direct effect of depression on self-efficacy × direct effect of self-efficacy on CLS2+]; p < 0.001). In model (iii) depression was associated with CLS with ≥ 2 partners indirectly through low self-efficacy for sexual safety (indirect Beta = 0.149; p < 0.001) and separately via higher levels of recreational drug use (indirect Beta = 0.050; p < 0.001). In model (iv) depression was associated with CLS with ≥ 2 partners indirectly through low self-efficacy for sexual safety and then higher levels of recreational drug use on the same pathway (indirect Beta = 0.076; p < 0.001). Path coefficients are presented below for model (iii) since it had the best model fit (Table 2).

**Table 2:** Comparing model fit indices of conceptual models with differing causal pathways between depressive symptoms and CLS with ≥ 2 partners

Models (in order of better model fit)^a^	RMSEA	CFI	TLI
Model (iii)	0.047 [0.044, 0.050]; p = 0.932	0.963	0.958
Model (i)	0.049 [0.046, 0.052]; p = 0.779	0.961	0.955
Model (iv)	0.053 [0.050, 0.056]; p = 0.056	0.953	0.947
Model (ii)	0.054 [0.051, 0.057]; p = 0.019	0.952	0.945
Model (v)	0.054 [0.051, 0.057]; p = 0.010	0.951	0.944

^a^Ranked according to the RMSEA first-although relative model fit statistics (information criterion statistics; AIC, BIC, and ABIC) do exist and are commonly used for model comparison, these indices cannot be estimated for models with categorical/binary variables, which use a weighted least squares estimator

### Path Coefficients in Conceptual Model (iii)

**Fig. 3:**
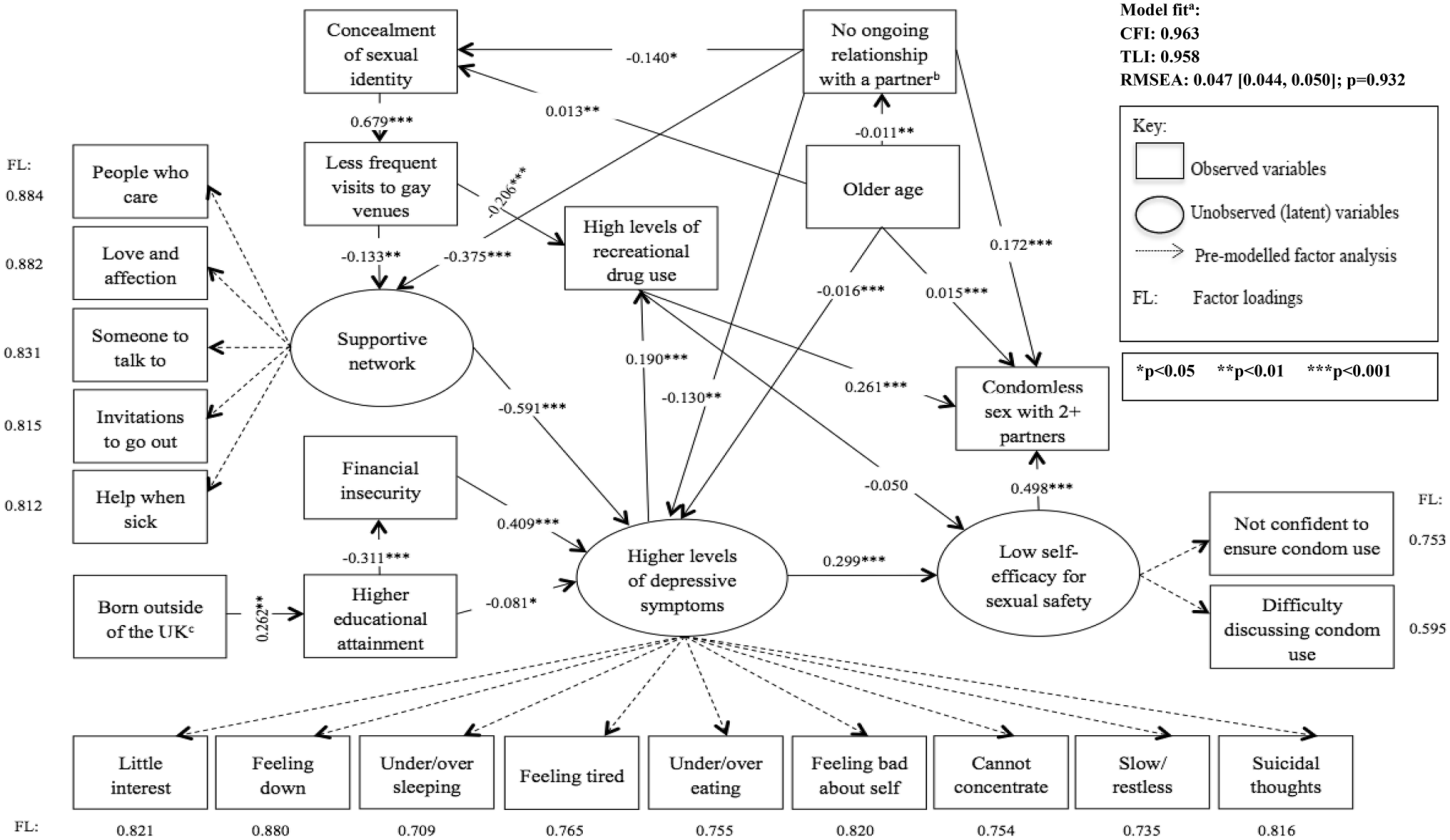
SEM of conceptual model (iii) of the link between depression and CLS with ≥ 2 partners in the AURAH study. ^a^The model is considered to have a satisfactory fit if: CFI and TLI are ≥ 0.90 & RMSEA is ≤ 0.08. The model is considered to have a good fit if: CFI and TLI are ≥ 0.95 & RMSEA is ≤ 0.06 (higher 90% CI ≤ 0.08), p > 0.05 [37, 45]. ^b^The gender of the partner was not specified. Men who had a female partner may have been less likely to disclose their sexual orientation, explaining the negative coefficient with concealment of sexual identity (a positive coefficient was hypothesized). ^c^The majority of men attended a clinic in London (75.9%) and may represent a select group of migrants of higher socio-economic status seeking job opportunities, explaining the positive coefficient

Each of the three CFAs (for depressive symptoms, self-efficacy for sexual safety, and supportive network) terminated normally, with p-values < 0.001 for all factor loadings. Figure 3 presents the results of the SEM for conceptual model (iii), including factor loadings for each observed item. All pathways specified were significant (p-values < 0.05). For instance, higher levels of depressive symptoms were associated with low self-efficacy for sexual safety (Beta = 0.299; p < 0.001) and low self-efficacy for sexual safety was associated with CLS with ≥ 2 partners (Beta = 0.498; p < 0.001). Higher levels of depressive symptoms were associated with greater number of recreational drugs used (Beta = 0.190; p < 0.001) and greater number of recreational drugs used was associated with CLS with ≥ 2 partners (Beta = 0.261; p < 0.001). Of the factors investigated to be directly associated with depression, the largest Beta coefficient was for the inverse association with higher levels of a supportive network (Beta = -0.591; p < 0.001). The second largest was for financial insecurity (Beta = 0.409; p < 0.001). The largest Beta coefficient in the conceptual model for direct effects was for the association between concealment of sexual identity and less frequent visits to gay venues (Beta = 0.679; p < 0.001).

In terms of the indirect pathways specified, concealment of sexual identity was associated with depression indirectly through less frequent visits to gay venues and lower levels of a supportive network (indirect Beta = 0.054; p = 0.003). Concealment of sexual identity was associated with lower levels of recreational drug use indirectly via less frequent gay venue attendance (indirect Beta = − 0.140; p < 0.001). Higher educational attainment was associated with less depression indirectly through greater financial security (indirect Beta = − 0.127; p < 0.001). Finally, older age was associated with lower levels of recreational drug use via concealment of sexual identity and less frequent visits to gay venues (indirect Beta = − 0.002; p = 0.003). The indirect pathways from depression to CLS with ≥ 2 partners are described above. Of note, there was no direct association between depression and CLS with ≥ 2 partners in this model; when including an additional direct link between depression and CLS with ≥ 2 partners, the p-value for the direct effect was 0.444 (Beta = 0.034).

## Discussion

Presented in this paper is the novel use of SEM to investigate five conceptual models of hypothesized causal links between socio-economic factors, depression and other psychosocial measures, and CLS with ≥ 2 partners among GBMSM. Compared to traditional regression analysis, SEM allows investigation of (complex) mediation chains and examination of the validity of the entire hypothesized conceptual model. This paper extends previous findings of a direct link between clinically significant depressive symptoms and CLS among GBMSM in the AURAH study [21], as well as in a number of other studies [5–22].

There may be numerous complex mechanisms of association between depression and sexual risk taking. Three of the five conceptual models investigated in this study were consistent with the AURAH data. In particular, they were consistent with the hypothesis that self-efficacy for sexual safety may be an important intermediary factor on the causal pathway leading to sexual risk taking. In all models, the Beta coefficient for the association with CLS with ≥ 2 partners was greater for self-efficacy than for drug use and other socio-economic factors investigated. Removing the indirect effect via low self-efficacy for sexual safety, as was done in conceptual model (ii), substantially reduced the model fit. This is in line with previous U.S. studies of GBMSM [14, 35]. Nevertheless, findings in this study indicate that recreational drug use is also an important mediator, reiterating findings from a previous study of Canadian GBMSM [36]. In the current study, data were marginally more consistent with the hypothesis that depression is associated with CLS with ≥ 2 partners either by lowering one’s self-efficacy for sexual safety or by leading to higher levels of recreational drug use on a distinct pathway. This suggests there may be important reasons for recreational drug use among GBMSM with depression other than low self-efficacy for sexual safety, which triggers drug use in order to facilitate cognitive escape from the perceived risk of STI/HIV transmission. These alternative reasons may include the use of drugs to alleviate depressive symptoms via cognitive disengagement, including drug use to lessen feelings of inadequacy in order to enable more enjoyable sexual experiences. Drugs may also be used to cope with other stressors including those common to the general population, such as finances, as well as those unique to sexual minorities, such as homophobic discrimination and heteronormative discourse. Recreational drug use could then lead to sexual risk taking via autonomic or central nervous system mechanisms that decrease self-regulation. There may also be other factors, which were not collected in AURAH, that explain (i.e. confound), fully or partially, the association observed between depression and recreational drug use: genes that confer risk for the co-occurrence of depression and substance use disorders [48], personality traits associated with sensation-seeking/sexual compulsivity [49], childhood sexual abuse [50], intimate partner violence [50], and internalized homophobia [50, 51].

In terms of the overall conceptual model, older rather than younger age was found to be associated with CLS with ≥ 2 partners. This differs from previous studies in the UK [52] and other high-income countries [53]. Differences between studies may reflect different recruitment sites, geographic differences, and use of different measures of sexual behaviour. In the SEM, not being in an ongoing relationship was associated with CLS with ≥ 2 partners. This association has been investigated in one other UK study of GBMSM [54], which failed to find a link in unadjusted analysis.

The same associations with depression that were observed in previous studies of GBMSM in high-income countries were also found in the SEM in this study: younger age, markers of lower socio-economic status, lower levels of a supportive network [16, 55], recreational drug use [10], and concealment of sexual orientation [21]. The exception to this was the association observed with relationship status in SEM, which was unlike in previous studies of GBMSM [55, 56]. Although not being in an ongoing relationship with a partner was associated with lower levels of a supportive network that was in turn associated with more depression, there also appeared to be a direct association between no ongoing relationship with a partner and less depression. The SEM might provide us with additional information given simultaneous adjustment for hypothesized associations with a supportive network, relationship status, and depression. It may be that men with low levels of social support despite having an ongoing stable partner may suffer worse depressive symptomatology than men with low levels of social support and no stable partner, due to the profound burden associated with an unhappy partnership. Another possibility is that intimate partner violence may be occurring within the context of an ongoing relationship, which may in turn lead to depression [50, 57]. The impact of an ongoing relationship on depressive symptoms is clearly complex.

Findings from the SEM also provide further insight into the relationship between minority related stressors and depressive symptoms. In Meyer’s minority stress model, one source of stress related to coping with a sexual minority status is concealment of sexual identity, with implications for psychological functioning. Identifying as a sexual minority is thought to ameliorate stress through opportunities for affiliation, social support, and coping [51]. SEM findings provide evidence in support of this theory since concealment of sexual identity was associated with depression indirectly through less frequent visits to gay venues and lower levels of a supportive network. However, being ‘out’ was also associated with higher levels of recreational drug use indirectly through more frequent visits to gay venues in the SEM. It is possible that the health benefits of disclosing one’s sexual identity may be diminished for some men via the relatively pronounced/frequent use of drugs on the gay scene.

## Limitations

Given the cross-sectional design of the AURAH study it is not possible to be assured of the direction of associations. Therefore, the hypothesized causal sequence assessed in SEM may operate in the other direction, with implications for intervention. There is a need to design and conduct longitudinal studies powered to investigate depression and CLS (and STI/HIV incidence) among GBMSM, and to assess the conceptual model presented in this paper within the framework of SEM. In addition, there may be unmeasured confounding in this study.

Due to small numbers, it was not possible to stratify the sample and investigate the conceptual model separately for different identities under the umbrella of sexual minority status. This is important as gay-identified and plurisexual-identified men may have different needs and experiences. For instance, there is a growing body of evidence to suggest that bisexual-identified men experience a greater burden of depressive symptomatology than do gay-identified men [55]. It was not possible in this study to investigate the concept of intersectionality; whether men subject to multiple systems of oppression, on the basis of their sexual identity, ethnicity, and socio-economic status, were at increased risk of poor mental health symptoms [58].

The AURAH study is important in the context of GUM services but the findings cannot necessarily be generalized to all GBMSM in the UK. The use of non-probability sampling methods may also limit the external validity of the sample i.e. the men recruited for AURAH may not be representative of all GBMSM attending GUM clinics in the UK. Some attempts were made to improve representativeness of the sample; at study start, all sites were asked to identify different clinic days/times each week for recruitment.

In total, 5% of GBMSM in this sample reported ever having used pre-exposure prophylaxis (PrEP). It was not possible to ascertain whether CLS in the past 3 months occurred with or without PrEP treatment. With the PrEP Impact Trial in England and likely increase in awareness of PrEP in recent years, the prevalence of PrEP use has increased substantially since the time of the AURAH study. Future studies will need to account for PrEP use in the definition of CLS outcomes, and additionally investigate whether depression is associated with low self-efficacy for PrEP use. Studies also need to take into account knowledge of an HIV-positive partner’s HIV treatment status in defining CLS outcomes, as recent evidence has unequivocally shown that someone with an undetectable viral load cannot transmit HIV [59]. However, irrespective of these factors, CLS outcomes remain important in relation to risk of transmission of other STIs.

## Implications for Intervention

Among sexually active GBMSM attending GUM services, management of depression alongside interventions surrounding self-efficacy for sexual safety may play an important role in HIV/STI prevention. In order to provide individuals with the tools needed to exercise self-protective control over interpersonal sexual situations, it may be useful to offer a guided self-enablement programme that simulates experiences of mastery in the exercise of personal control [60–62]. A combination of efforts that address stress related to sexual minority status and socio-economic hardships common to the general population may be important factors in alleviation of depressive symptomatology. In particular, having a supportive network, which may be intricately linked to disclosure of one’s sexual identity and community affiliation, appears to play an important protective role with regards to depression among GBMSM. Integration of substance use services into GUM clinics or referral to such services may be highly effective in reducing future sexual risk taking. It could be suggested that interventions focused on younger men should address recreational drug use on the gay scene and interventions focused on older men should address relationship counselling, community participation, and social support.

## Conclusion

There may be numerous complex mechanisms leading from depression to sexual risk behaviour among GBMSM. Multipronged STI/HIV prevention programmes that address the causes of depression among GBMSM as well as self-efficacy for sexual safety and recreational drug use, may be useful in reducing sexual risk taking. Future studies of GBMSM should move beyond examining simple direct effects of depressive symptoms on CLS and examine hypothesized relationships in an SEM framework, in order to better understand mechanisms of effect and guide prevention interventions.

## Data Availability

We have a number of planned analyses for the AURAH study, but welcome proposals for additional analysis; please contact Dr Fiona Lampe (f.lampe@ucl.ac.uk). The Study Core Group will review proposals.
